# Synergistic Antifungal Activity of Berberine Derivative B-7b and Fluconazole

**DOI:** 10.1371/journal.pone.0126393

**Published:** 2015-05-19

**Authors:** Li Ping Li, Wei Liu, Hong Liu, Fang Zhu, Da Zhi Zhang, Hui Shen, Zheng Xu, Yun Peng Qi, Shi Qun Zhang, Si Min Chen, Li Juan He, Xin Ju Cao, Xin Huang, Jun Dong Zhang, Lan Yan, Mao Mao An, Yuan Ying Jiang

**Affiliations:** 1 Shanghai Tenth People's Hospital, and Department of Pharmacology, Tongji University School of Medicine, 1239 Siping Road, Shanghai, 200092, PR China; 2 New Drug Research and Development Center, School of Pharmacy, Second Military Medical University, Shanghai, China; Louisiana State University, UNITED STATES

## Abstract

Our previous study demonstrated berberine (BBR) and fluconazole (FLC) used concomitantly exhibited a synergism against FLC-resistant *Candida albicans in vitro*. We also suggested BBR played a major antifungal role in the synergism of FLC and BBR, while FLC increased intracellular BBR concentrations. Our following systematic structural modification and reconstruction of BBR core identified the novel scaffold of *N*-(2-(benzo[*d*][1,3]dioxol-5-yl)ethyl)-2-(substituted phenyl)acet-amide derivatives 7a-i, including B-7b and B-7d exhibiting remarkable synergistic antifungal activity and low cytotoxicity. Here, the study mainly investigated the synergistic activity of FLC and B-7b and the underlying mechanism. *In vitro* interaction of FLC and B-7b was investigated against 30 FLC-resistant clinical isolates of *C*. *albicans* and non-*C*. *albicans* species, including *Candida tropicalis*, *Candida parapsilosis*, *Candida glabrata*, *Candida krusei* and *Cryptococcus neoformans*. The potent synergistic activity of B-7b in combination with FLC against FLC-resistant *C*. *albicans* was found through the checkerboard microdilution assay. The findings of agar diffusion tests and time-kill curves confirmed its better synergism with FLC. And as expected, B-7b exhibited much lower cytotoxicity than BBR to human umbilical vein endothelial cells. In contrast to BBR, we found that endogenous ROS augmentation was not involved in the synergism of FLC and B-7b. According to the results from our present comparative proteomic study, it seemed that the disruption of protein folding and processing and the weakening of cells’ self-defensive ability contributed to the synergism of FLC and B-7b. Together, these results suggested novel scaffold BBR derivative B-7b could be a promising synergist in combination with FLC for the treatment of invasive fungal infections.

## Introduction

*Candida albicans*, one of prevalent human fungal pathogens, mainly causing superficial mycoses, invasive mucosal infections, and disseminated systemic disease, is still the most common pathogenic fungus, and the infected patients have a mortality rate of approximately 40%, although an increase in the frequency of infections due to non-*C*. *albicans* species, including *Candida tropicalis*, *Candida parapsilosis*, *Candida glabrata*, *Candida krusei* and *Cryptococcus neoformans* [1–11]. In spite of the need for effective antifungal therapy is increasing, the available antifungal agents are still limited. Fluconazole (FLC) is most widely used due to its high bioavailability and low toxicity [12,13]. However, with the increasing clinical use of FLC, drug-resistant isolates are emerging rapidly, which have significantly limited the effectiveness of FLC and contributed to the failure of its treatment for *C*. *albicans* infections in the clinic [14,15].

Berberine (BBR), an alkaloid widely found in plant families including *Hydrastis canadensis* (goldenseal), *Berberis aquifolium* (Oregon grape), and *Berberis vulgaris* (barberry), is currently demonstrated to have antimicrobial activity against different kinds of organisms such as bacteria, viruses, protozoans and fungi, and have multiple clinical uses including antidiarrheic, antiinflammatory, antiarrhythmic and anticancer [16–21]. Its synergistic antifungal properties in combination with some known antifunal agents (such as FLC, amphotericin B and miconazole) have also been reported [22–24]. The better-established synergistic combinations of BBR with azoles help to enhance the antifungal activities of azoles, especially for FLC used as first-line drug against candidiasis, and therefore the investigation of the *in vitro* interaction between natural antimicrobial (e.g. BBR) and synthetic chemical therapeutic agent (e.g. FLC) contribute to the development of new antifungal therapeutics [25,26].

We have demonstrated that BBR and FLC used concomitantly is highly efficacious in killing FLC-resistant *C*. *albicans* clinical strains [27], and BBR plays a crucial role in the synergistic antifungal activity of FLC and BBR, while the role of FLC is to assist BBR in accumulating in *C*. *albicans* cells, especially in the nucleus, where BBR probably binds to DNA, causing cell cycle arrest and DNA damage, as described in detail previously [28]. Our further proteomic study suggested that increased generation of endogenous reactive oxygen species (ROS) and mitochondrial aerobic respiration shift contributed to the synergistic activity of FLC and BBR against FLC-resistant *C*. *albicans* [29].

However, BBR itself is not a good synergist to be used in combination with FLC because of its high toxicity [30,31]. As described in detail previously [32], we carried out a series of systematic structural modification and reconstruction of BBR core, aiming to seeking novel synergistic agents with lower cytotoxicity to improve the effectiveness of FLC against FLC-resistant *C*. *albicans*, and identified the novel scaffold of N-(2-(benzo[*d*][1,3]dioxol-5-yl)ethyl)-2-(substituted phenyl) acetamides such as B-8, B-7b and B-7d.

It is hypothesized that the novel scaffold especially B-7b, when combined with FLC, exerts potent synergistic antifungal activity against FLC-resistant *C*. *albicans* and other yeast fungi. In this study, selected BBR derivatives were tested for their ability to enhance the antifungal efficacy of FLC by time-kill curves, agar diffusion test and checkerboard microdilution assay. In addition, a comprehensive comparative proteomic analysis was performed to investigate the synergistic mechanism between FLC and B-7b.

## Materials and Methods

### Strains

Thirty clinical isolates of FLC-resistant *C*. *albicans*, one FLC-sensitive *C*. *albicans* SC5314, one *C*. *neoformans* 56992, *C*. *tropicalis* ATCC20026, *C*. *parapsilosis* ATCC 22010, *C*. *krusei* ATCC2340 and *C*. *glabrata* ATCC1182 provided by professor Changzhong Wang (School of integrated traditional and western medicine, Anhui university of traditional Chinese medicine, Hefei, China) were used in this study. All strains were maintained on SDA agar (1% peptone, 4% dextrose, and 1.8% agar) plates and re-cultured at least monthly from -80°C stock. For use in the experiments, yeast-phase cells of the various strains were grown YPD broth overnight in a rotary shaker at 30°C.

### Agents

**Fig 1:**
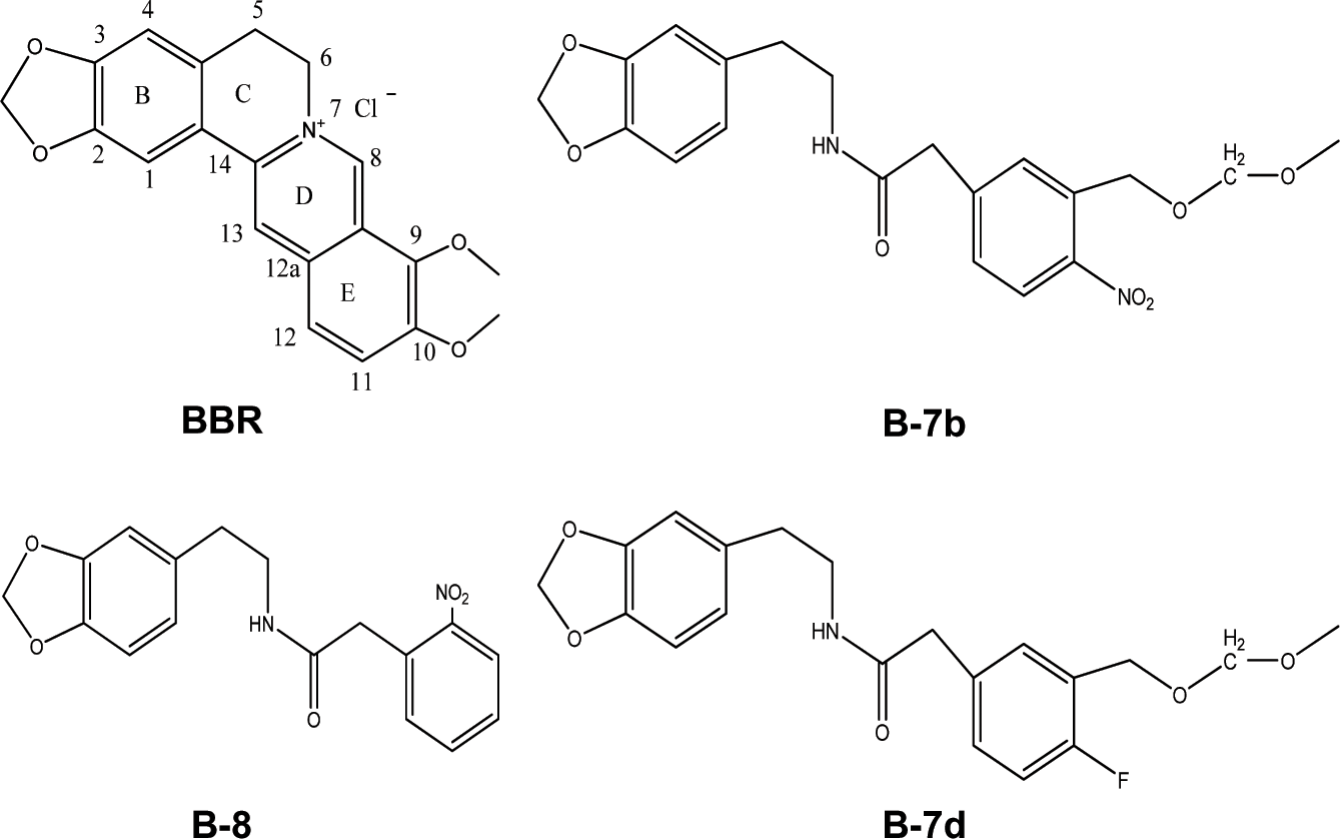
The structures of BBR and BBR derivatives (B-8, B-7b, B-7d).

Drugs prepared in dimethyl sulfoxide (DMSO) included FLC (Pfizer-Roerig Pharmaceuticals, New York, NY), BBR (Sigma-Aldrich, St. Louis, MO) and BBR derivatives B-8, B-7b and B-7d (Fig 1) structured and identified by methods shown in [32], and their initial stored concentration was 6.4 mg/ml in DMSO [27].

### Checkerboard microdilution assay

The in vitro MICs of the compounds against all 30 clinical isolates of *C*. *albicans* were determined by the microbroth dilution method according to the Clinical and Laboratory Standards Institute (formerly the National Committee for Clinical Laboratory Standards) as described previously [27]. The initial concentration of fungal suspension in RPMI 1640 medium was 10^3^ CFU/ml, and the final concentrations ranged from 0.125 to 64 μg/ml for FLC and 1 to 32 μg/ml for B-7b. The final concentration for FLC or B-7b alone ranged from 0.125 to 64 μg/ml. 96-well flat-bottomed microtitration plates were incubated at 35°C for 24 h or 72 h. Optical density was measured at 630 nm. MIC_80_ were determined as the lowest concentration of the drugs (alone or in combination) that inhibited growth by 80%, compared with that of drug-free wells.

The data obtained by the checkerboard microdilution assays were analyzed using the model-fractional inhibitory concentration index (FICI) method based on the Loewe additivity theory. The fractional inhibitory concentration index (FICI) is defined as the sum of the MIC of each drug when used in combination divided by the MIC of the drug used alone. Synergy and antagonism were defined by FICIs of ≤0.5 and >4, respectively. An FICI result of >0.5 but ≤4 was considered indifferent [33].

### Agar diffusion test

*C*. *albicans* 103 (one FLC-resistant isolate with a MIC of >64 μg/ml for B-7b) was tested by agar diffusion assay as described previously [27]. A 3-ml aliquot of 10^6^-CFU/ml suspension was spread uniformly onto the yeast extract-peptone-dextrose agar plate with or without 4μg/ml FLC. Then, 6-mm paper disks impregnated with FLC and B-7b alone or in combination were placed onto the agar surface. There was 5μl of DMSO in control disks. Inhibition zones were measured after incubation at 35°C for 48 h.

### Time-kill test

*C*. *albicans* 103 in RPMI 1640 medium was prepared at the starting inoculum of 10^3^ CFU/ml or 10^5^ CFU/ml. The concentrations were 4 μg/ml of B-7b and 8 μg/ml of FLC. DMSO comprised <1% of the total test volume. At predetermined time points (0, 4, 8, 12, 16, 24, 36 and 48 h) after incubation with agitation at 35°C in a shaking incubator, 100-μl aliquot was removed from every solution and serially diluted 10-fold in sterile water. A 100-μl aliquot from each dilution was spread on the sabouraud dextrose agar plate. Colony counts were determined after incubation at 35°C for 48 h. Synergism and antagonism were defined as a respective increase or decrease of ≥2 log_10_ CFU/ml in antifungal activity produced by the combination compared with that by the more active agent alone after 24 h, while a change of <2 log_10_ CFU/ml was considered indifferent [27].

### Cytotoxicity evaluation of BBR derivatives using XTT assay

Human umbilical vein endothelial cell (HUVEC) was cultured in DMEM medium (HyClone) supplemented with 10% fetal bovine serum (HyClone). The cytotoxic effect of B-7b on HUVEC viability was assessed by the XTT assay [34]. Briefly, cells (3×10^3^ cells/well) were cultured in 96-well microtiter plates and treated with different concentrations of B-7b for 24 h. At the end of incubation, 50 μL of PMS-XTT solution (final concentration, 50 μg of XTT and 0.38 μg of PMS per well) was added to each well and incubated at 37°C for 4 h. Absorbance at 450 nm was measured using the Eliza Plate Reader.

### Protein sample preparation and NanoLC-MS/MS

FLC-resistant *C*. *albicans* 103 cells (OD_600_ = 0.1) were treated or untreated with FLC (64μg/ml) and/or B-7b (16 μg/ml) for 9 h at 30°C. Cells were harvested and washed with phosphate-buffered saline (PBS, pH 7.4) buffer. Next, the cell pellet was lysed in 10 ml of lysis buffer [50 mM Tris, 1.5 mM EDTA, 1% (v/v) Triton×100, 0.4% (w/v) SDS, pH 7.5] and 20 ml of 0.5 mm diameter glass beads (Biospec, Bartlesville, OK) by vortexing for 30 seconds and cooling on ice for 30 seconds repeatedly in a Mini Bead-beater (Biospec) until at least 80% of the cells had been lysed as determined by phase-contrast microscopic examination. After centrifugation at 13 000 g for 5 min, the clarified protein supernatant was determined using the RC DC Protein Assay Kit (Bio-Rad, Herclues, CA) and according to the manufacturer’s directions.

The above obtained protein supernatant was applied to an EASY-nano liquid chromatography (LC) system (Proxeon Biosystem) coupled online to an ESI-LTQ-Orbitrap Velos mass spectrometer (Thermo Fisher Scientific) as a previously described method [35]. The database searches were performed with the following parameters: Candida Genome Database (CGD), *C*. *albicans* SC5314_version_A22-s04-m01-r04_orf_trans_all.fasta. Protein abundance was calculated from all qualified corresponding peptides matched to that protein and final results were filtered using a 1% false discovery rate (FDR).

### Bioinformatic analysis

To investigate biological significance, the identified differential proteins in each comparison were input into DAVID bioinformatics resources 6.7 (the Database for Annotation, Visualization, and Integrated Discovery, http://david.abcc.ncifcrf.gov/) for functional annotation and KEGG (the Kyoto Encylopedia of Genes and Genomes) pathway analysis [36]. Briefly, DAVID functional annotation and KEGG pathway analysis were performed on the list of differential proteins with differential ratio of over ±1.2 and a nominal p-value of less than 0.05 in each comparison. For the Gene Ontology (GO) enrichment in DAVID, the GO terms of “Biological Process”, “Cellular Component” and “Molecular Function” were used, and only the terms that reported a p-value of ≤0.05 and count number ≥5 proteins were selected. The signaling pathways were identified and mapped using DAVID and the KEGG Automatic Annotation Server (KAAS) [37]. The KEGG pathways with at least 5 proteins and p-value of <0.05 were identified as enriched.

Further, protein-protein interaction (PPI) networks of the differential proteins were predicted and analyzed using STRING v9.1 (Search Tool for the Retrieval of Interacting Genes) database [38]. STRING, a web resource (http://string-db.org/) dedicated to protein-protein interactions including direct (physical) and indirect (functional) associations, quantitatively integrates information from numerous sources including genomic context, high-throughput experiments, (Conserved) co-expression and previous knowledge. Known and predicted protein-protein interactions are scored and integrated, resulting in comprehensive protein networks currently covering 5,214,234 proteins from 1133 organisms in the STRING database. This analysis gave uniquely comprehensive coverage of both experimental and predicted interaction information with a relative confidence score, implying that only interactions with high level of confidence were extracted and considered as valid links for the PPI network [38–40]. In a given network, proteins are represented as nodes and the interactions are defined as edges (lines), and thicker edges represent stronger associations. For a PPI network, the majority of the nodes were linked with each other, while some of altered proteins were isolated without partners. The “hub” proteins were proteins with a number of edges, meaning the capability of physically interacting with many partners.

### Statistical analysis

At least three biological replicates were performed for all experiments unless otherwise indicated. Analysis of variance (ANOVA) was used when multiple groups were analyzed and the two-tailed Student’s t-test was used for analysis of two groups, with paired analysis when appropriate. Statistical significance was set at a p value of 0.05, 0.01 or 0.001, indicated by *, **, ***, respectively.

## Results

### Comparison of the activities of three BBR derivatives and BBR against FLC-resistant *C*. *albicans*

**Fig 2:**
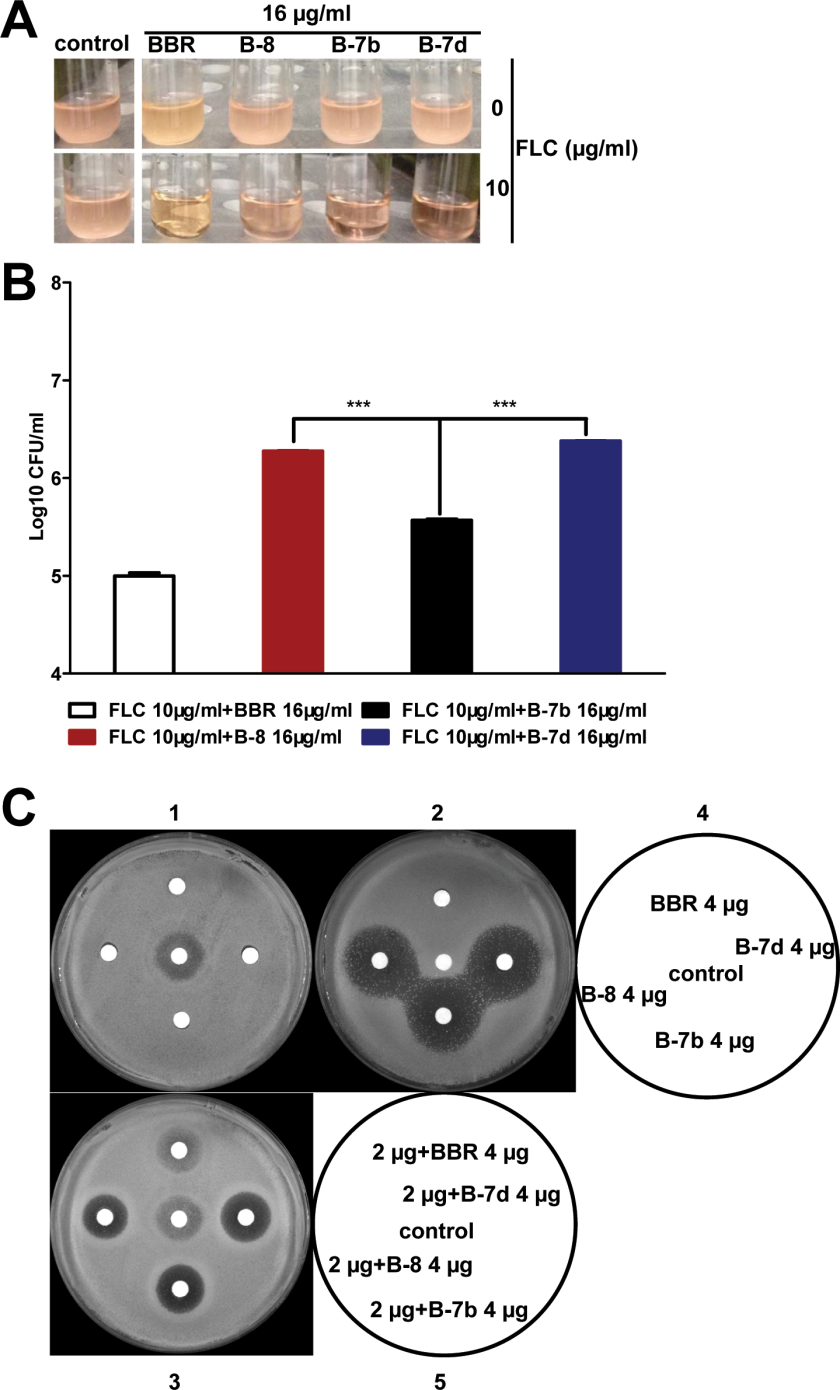
Growth condition of *C*. *albicans* 103 treated with FLC, BBR and different BBR derivatives. Exponetially growing FLC-resistant *C*. *albicans* 103 were treated with or without FLC (10 μg/ml), BBR (16 μg/ml) and different BBR derivatives (16 μg/ml B-8, B-7b, B-7d) alone or the combinations of FLC and BBR or BBR derivatives in a shaking incubator. (A) Pictures of the growth condition of *C*. *albicans* with an initial inoculum of 10^5^ CFU/ml were taken after 24 h incubation. (B) The growth change of *C*. *albicans* 103 with an initial inoculum of 10^5^ CFU/ml after 24 h. Aliquots were obtained at 24 h and serially dilutions were spreaded on agar plates. Colony counts were determined after 48 h incubation. (C) Agar disk diffusion assay of different compounds (BBR, B-8, B-7b and B-7d) combined with FLC against *C*. *albicans* 103. Panels 1 and 3 show agar plates, and panel 2 shows an agar plate containing 4 μg/ml FLC. Panel 4 describes the images for panels 1 and 2, which containing 4 μg of BBR, B-8, B-7b, B-7d and 2 μg of FLC or just DMSO as control per disc. Panel 5 describes the image for panel 3, the combination of 4 μg compounds (BBR, B-8, B-7b and B-7d) with FLC (2 μg) or FLC (2 μg) alone as control were contained in each disc.

The highly effective *in vitro* synergism of FLC and BBR against FLC-resistant clinical isolates of *C*. *albicans* has been verified [27]. To seek whether N-(2-(benzo[*d*][1,3]dioxol-5-yl)ethyl)-2-(substituted phenyl) acetamide derivatives have the same antifungal effectiveness as BBR, We performed a comparison of BBR and the three BBR derivatives (B-8, B-7b and B-7d) against FLC-resistant *C*. *albicans* 103 by different methods. At a concentration of 16 μg/ml for BBR and BBR derivatives in the time killing assay, as shown in Fig 2A, the clarity of suspension culture treated with B-7b and FLC (10 μg/ml) after 24 h, was obviously clearer than those of the other two compounds. The statistical analysis for colony counts indicated that B-7b had better synergistic activity than the other two compounds in combination with FLC (10 μg/ml) against *C*. *albicans* 103 with the initial inoculum of 10^5^ CFU/ml, though weaker than that of BBR (Fig 2B).

Further to visualize their synergistic antifungal effect, all compounds and the combination with FLC (4 μg/ml) were analyzed by agar diffusion tests (Fig 2C). All compounds (BBR, B-8, B-7b and B-7d) had no antifungal activity in small amounts at 4 μg alone (Fig 2C1). At 4 μg, BBR combining with FLC showed no synergistic antifungal activity. In contrast, the three BBR derivatives showed potent synergistic antifungal effects on the agar plate with 4 μg/ml FLC (Fig 2C2). The mean diameters of the inhibitory zones for BBR, B-8, B-7b and B-7d were 0, 25.2, 27.0 and 26.3 mm, respectively. In addition, as shown in Fig 2C3, the sizes of the inhibitory zones were 0, 17.1, 17.6 and 17.6 mm around disks impregnated with 2 μg FLC plus compounds (BBR, B-8, B-7b and B-7d), respectively. In small amounts (such as 4 μg), B-7b had slightly better (or the same) synergistic antifungal activity compared with B-8 and B-7d, but much better than BBR. Therefore, we conducted all subsequent studies with B-7b as a representative example.

### In combination with FLC, B-7b at lower concentrations exhibits better synergistic activity than BBR against FLC-resistant *C*. *albicans*

**Fig 3:**
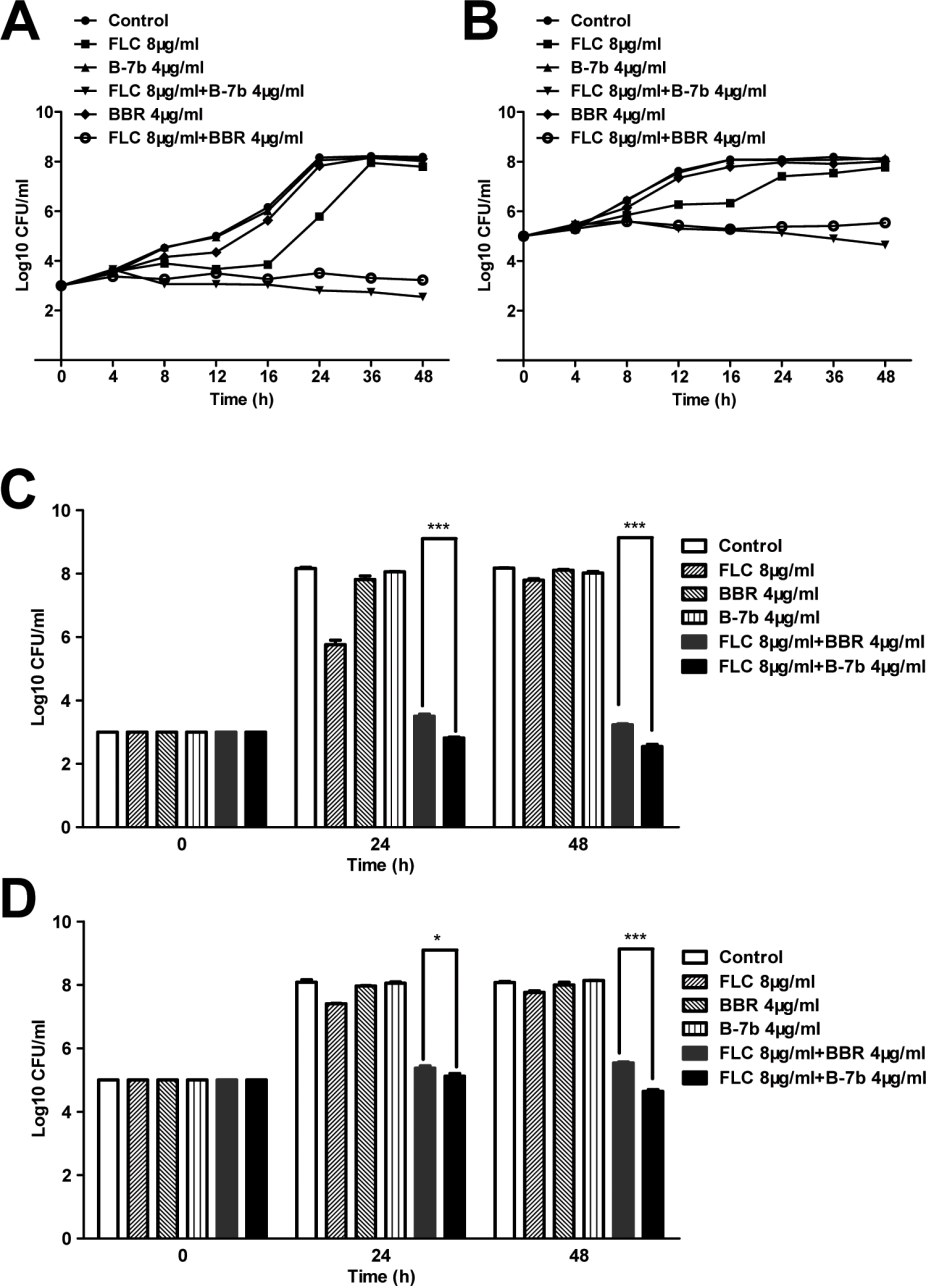
Time killing curves of *C*. *albicans* 103 treated with different concentrations of B-7b, BBR and FLC. FLC-resistant *C*. *albicans* 103 were treated with FLC (8 μg/ml), B-7b (4 μg/ml), BBR (4 μg/ml), FLC+ B-7b (8+4) μg/ml and FLC+ BBR (8+4) μg/ml by using initial inoculums of 10^3^ CFU/ml (A, C) and 10^5^ CFU/ml (B, D). Aliquots were obtained at the indicated time points and serially dilutions were spread on agar plates. Colony counts were determined after 48h incubation.

As the above description, in small amounts at 4 μg, B-7b had more powerful synergistic effect than BBR (Fig 2C). In order to determine whether B-7b at lower concentrations in combination with FLC showed better synergistic activity than that of BBR against FLC-resistant *C*. *albicans* 103, their synergism was also conμrmed by time-killing test (Fig 3). BBR (4 μg/ml), B-7b (4 μg/ml) or FLC (8 μg/ml) alone had no effect on the growth of *C*. *albicans* 103 after 48 h. However, the antifungal activity of FLC was dramatically raised by addition of BBR or B-7b. Under the initial inoculum of 10^3^ CFU/ml (Fig 3A), the combination of FLC with BBR or B-7b respectively produced a 4.56 or 5.24-log_10_-CFU/ml decrease compared with the number of CFU produced by 8 μg/ml of FLC alone at 48 h (Table 1). Given the beginning inoculum of 10^5^ CFU/ml (Fig 3B), the FLC+BBR or FLC+B-7b combination yielded 2.22 or 3.12-log_10_-CFU/ml decrease compared with the FLC alone at 48 h (Table 1). Despite of the starting inoculum, the combination of FLC and B-7b yielded a significant decrease of CFU/ml compared with the combination of FLC and BBR at both 24 h and 48 h (Fig 3C and 3D). Interestingly, the significance level for the synergistic effect of FLC and B-7b compared with that of BBR was observed earlier (at 24 h) and much more prominent (p <0.001), when the initial inoculum declined from 10^5^ CFU/ml to 10^3^ CFU/ml (Fig 3C and 3D).

**Table 1:** Decrease in log_10_ CFU/ml of C.albicans 103 using BBR or B-7b combining with FLC at 24 h or 48 h.

	Mean (±SD) Decrease in log_10_ CFU/ml of C.albicans 103 using BBR or B-7b combining with FLC at 24 h and 48 h.			
	10^3^CFU/ml		10^5^CFU/ml	
	24h	48h	24h	48h
FLC 8μg+BBR 4μg	2.26 (0.21)	4.56 (0.07)	2.03 (0.06)	2.22 (0.02)
FLC 8μg+B-7b 4μg	2.95 (0.17)	5.24 (0.02)	2.28 (0.07)	3.12 (0.005)

**Fig 4:**
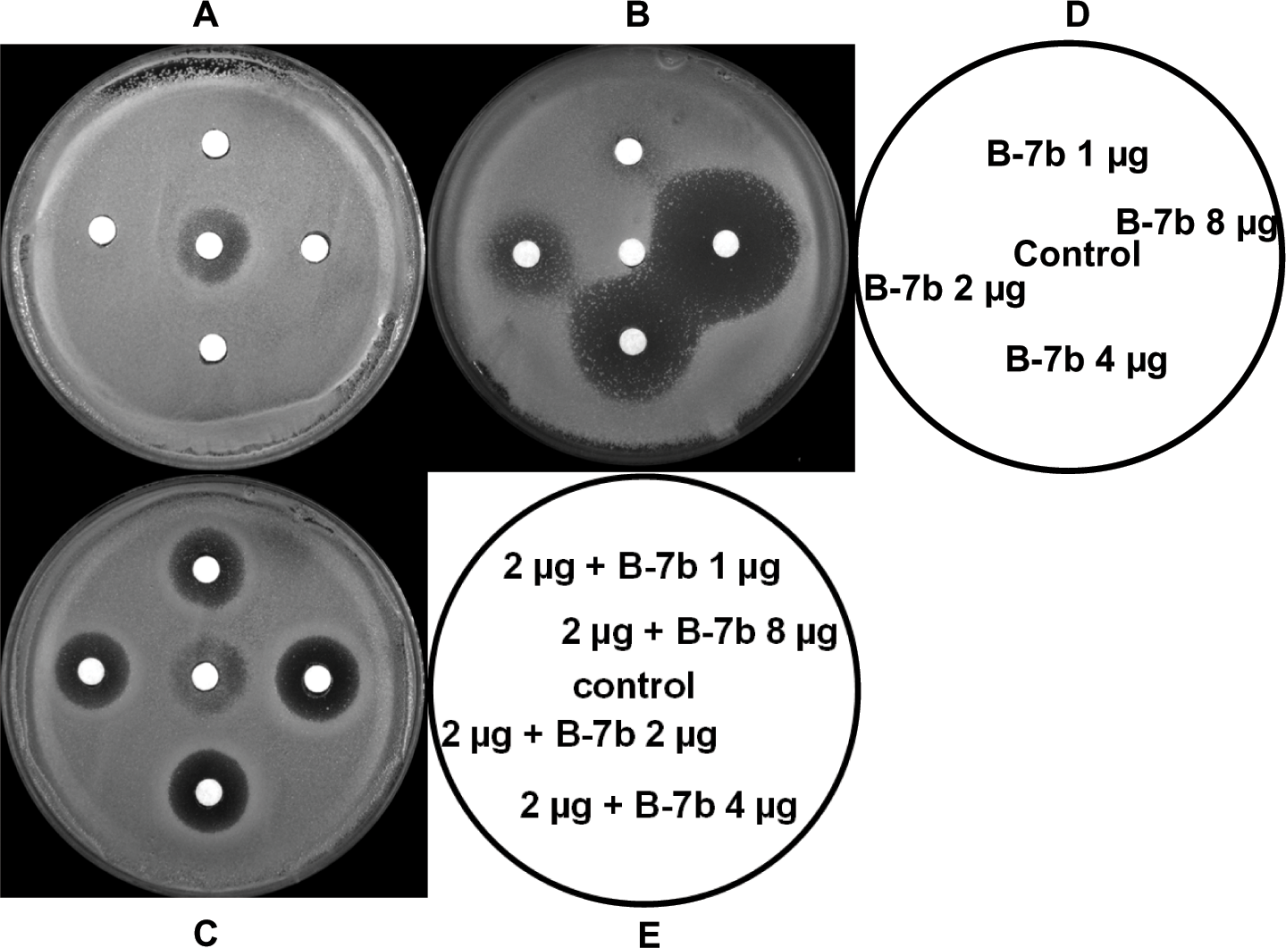
Agar disk diffusion assay of different concentrations of BBR derivatives combined with FLC against *C*. *albicans* 103. Agar disk diffusion assay of different concentrations of B-7b combined with FLC against *C*. *albicans* 103. Panels A and C show agar plates, and panel B shows an agar plate containing 4 μg/ml FLC. Panel D describes the images for panels A and B, which containing 1, 2, 4, 8 μg of B-7band 2 μg of FLC or just DMSO as control per disc. Panel E describes the image for panel C, the combination of B-7b (1, 2, 4, 8 μg) with FLC (2 μg) or FLC (2 μg) alone as control were contained in each disc.

To investigate the lower dosage of B-7b which in combination with FLC still exhibits its synergism, different concentrations of B-7b, FLC and the combination of B-7b and FLC (2 μg) were analyzed by agar disk diffusion assay as described in detail previously [27]. B-7b alone at 8, 4, 2, 1 μg per disc had no antifungal activity against the FLC-resistant *C*. *albicans* 103 (Fig 4A). While FLC at 2 μg per disc showed only weak inhibition effect against *C*. *albicans*, the halo surrounding the disc was cloudy with colony (Fig 4A and 4C). B-7b at 2μg still showed potent synergistic antifungal effect on the agar plate containing 4 μg/ml FLC (Fig 4B). The mean diameters of the inhibitory zones for 1, 2, 4 and 8 μg B-7b were 0, 16.7, 27.8 and 33.3 mm, respectively. Furthermore, when different amounts of B-7b (1, 2, 4 and 8 μg) was combined with FLC (2 μg), the clear halos surrounding the disc were observed, and the mean diameters of these zones were 15.0, 15.6, 17.8 and 18.3 mm, respectively.

### B-7b has much lower cytotoxicity when compared to BBR *in vitro*

**Fig 5:**
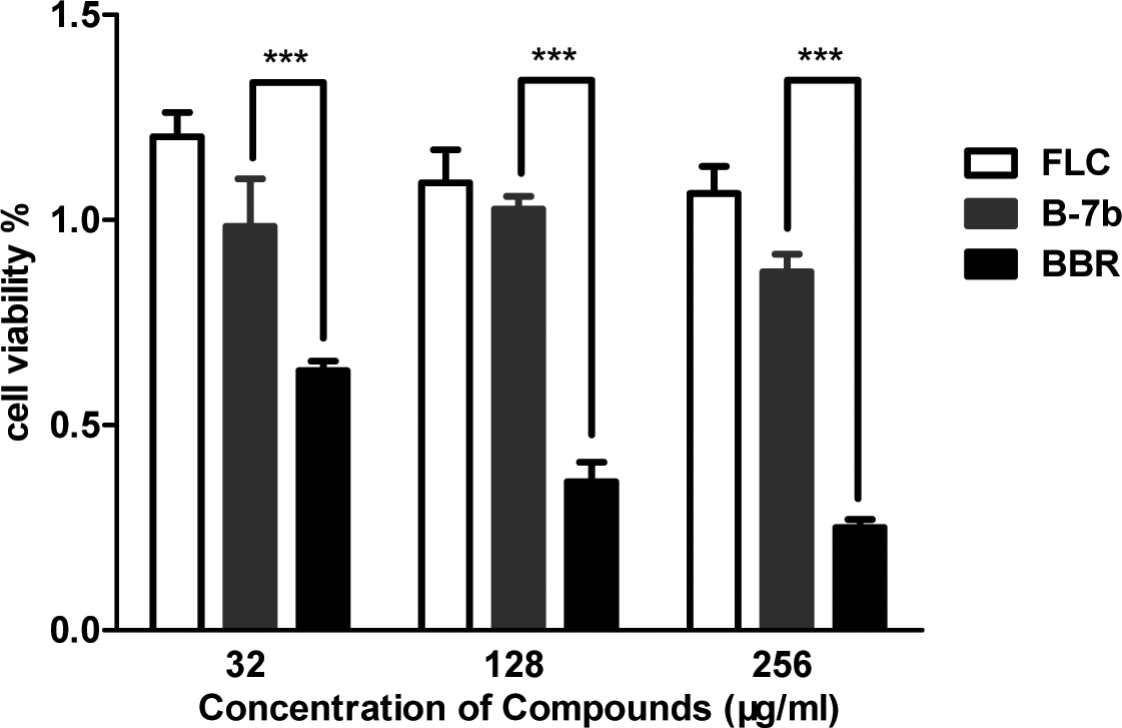
The in vitro cytotoxicity evaluation of B-7b using XTT assay. The cytotoxic effect of B-7b towards HUVEC viability was assessed by an XTT test following a 4-h treatment, when compared to that of FLC and BBR. ***, P<0.001 versus cells treated with BBR.

**Fig 6:**
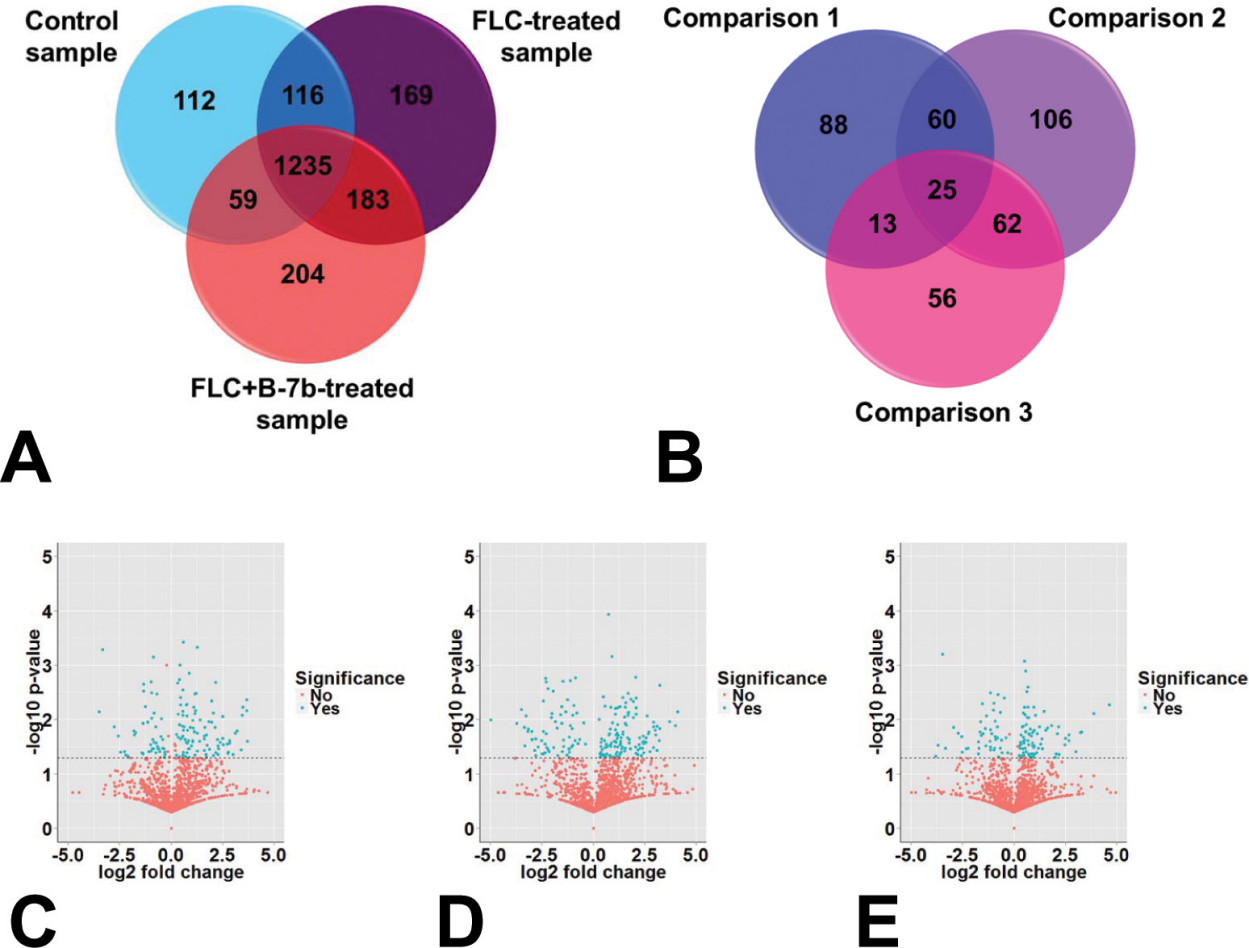
The summary of total identified proteins and differential proteins. (A) Venn diagram for the number of proteins overlapping among three samples: Control sample, FLC-treated sample treated by FLC 64μg/ml, and FLC+B-7b-treated sample treated by FLC 64μg/ml+B-7b 16μg/ml. (B) Venn diagram for differential proteins in paired comparisons. Comparison 1: differential proteins identified in FLC-treated sample versus Control sample; Comparison 2: differential proteins identified in FLC+B-7b-treated sample versus Control sample; Comparison 3: differential proteins identified in FLC+B-7b-treated sample versus FLC-treated sample. (C-E) Volcano plots show differential proteins (differential ratio of over ±1.2 and *p* < 0.05) in Comparison 1, 2 and 3.

**Fig 7:**
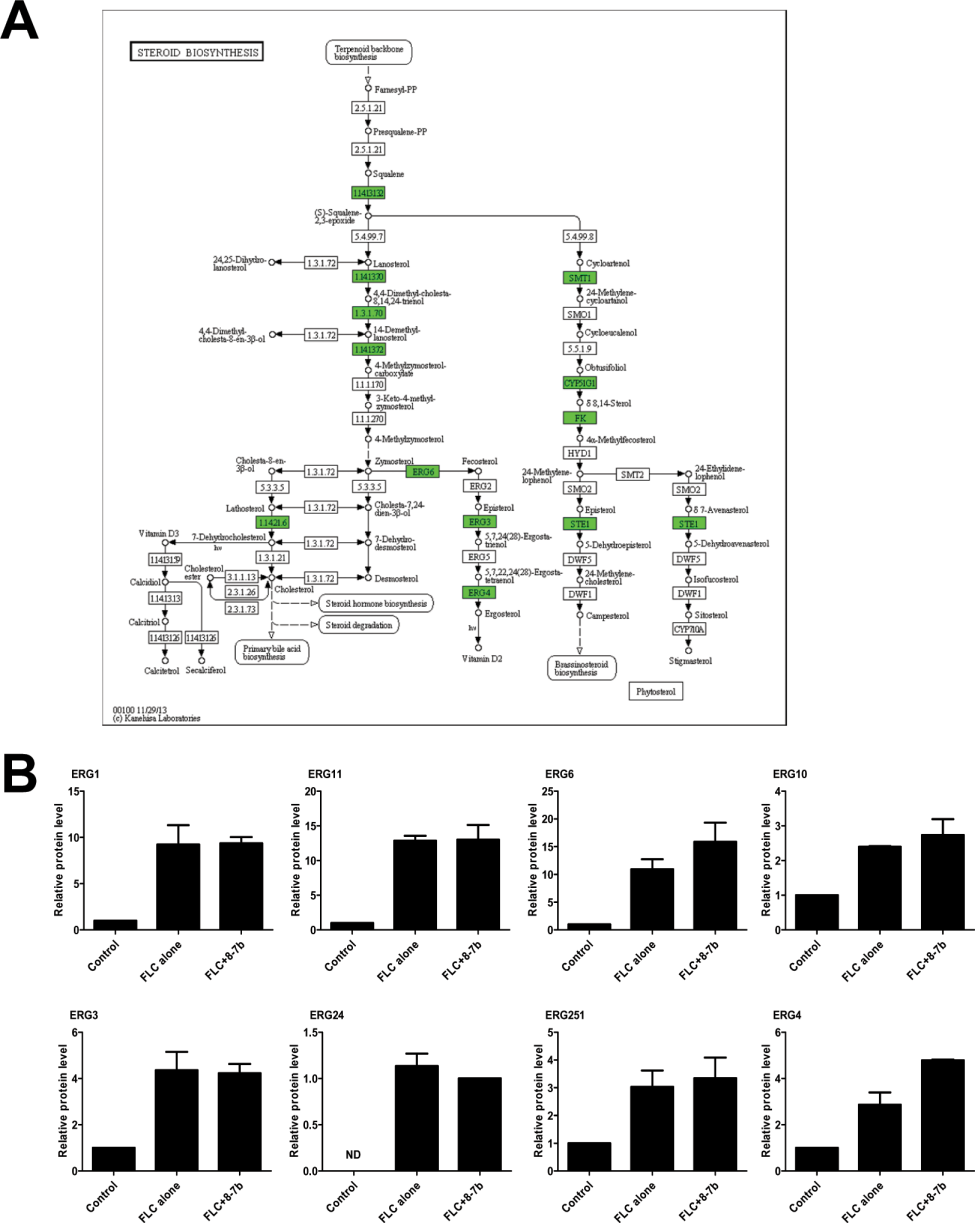
The significant steroid biosynthesis pathway (p<0.05) for differential proteins and the differential protein level in Comparison 1 and 2. (A) For each rectangular shape, green shapes indicate significantly up-regulated proteins. KEGG pathway analysis figure depicts the role or involvement of a specific protein or a family of related proteins at a particular location in the pathway. (B) The relative protein level of eight differential proteins compared to Control, corresponding to green rectangular shapes in A.

It is necessary to assess the toxicity of B-7b because of the high toxicity of BBR itself [30, 31]. In the study, *in vitro* cytotoxic effect of B-7b against HUVEC was performed via XTT assays. As shown in Fig 5, B-7b had no toxic effect on HUVEC at the concentration of 128 μg/ml, while BBR significantly (p <0.001) reduced cell viability (cell viability <50%) at concentrations ≥128 μg/ml. the results indicated that B-7b showed much lower toxicity and was quite promising when compared to BBR.

### The combination of FLC and B-7b against clinical FLC-resistant *C*. *albicans*

Due to the strong synergistic activity in combination with FLC when against FLC-resistant *C*. *albicans* 103, we further confirmed the synergistic activity of B-7b in combination with FLC against 30 clinical strains of FLC-resistant *C*. *albicans*. The MIC results of B-7b alone or in combination with FLC against 30 clinical FLC-resistant *C*. *albicans* tested by Checkerboard microdilution assay were summarized in Table 2. The MICs of B-7b or FLC alone against all tested strains were >64 μg/ml, respectively. However, the combination of FLC and B-7b markedly reduced the MIC_80_s, and the median FICI was 0.016 (range, 0.012 to 0.07), while the median FICI of BBR+FLC was 0.034 (range, 0.017 to 0.127) as shown in the previous report [27]. B-7b in combination with FLC displayed powerful synergism in all 30 FLC-resistant *C*. *albicans* (100%) tested, according to the analysis of FICI method.

**Table 2:** MICs of B-7b alone and in combination with FLC against 30 clinical *C*.*albicans*.

	MIC80(μg/ml)	
	median	range
FLC	>64	>64
B-7b	>64	>64
FLC/B-7b^a^	1/1	0.5-8/1-2
FIC index	0.016	0.012–0.07
interaction effect (n/%)^b^	Syn(30/100)	

^a^ MIC in combination expressed as [FLC]/[B-7b]. FLC: Fluconazole.

^b^ Syn, synergism. The number of strains and percentage for the interaction effect were shown.

### The combination of FLC and B-7b against varied yeast strains

We also tested antifungal activity of B-7b alone or in combination with FLC in other yeast strains (FLC-sensitive *C*. *albicans*, *C*. *neoformans*, *C*. *tropicalis*, *C*. *krusei*, *C*. *parapsilosis* and *C*. *glabratas*) (Table 3). The range of MICs of B-7b tested alone against all these strains were still >64 μg/ml. When in combination with FLC, the MICs of B-7b ranged from 1 μg/ml to 32 μg/ml. In *C*. *glabratas* and *C*. *krusei*, no synergistic effect was shown. However, the MIC of FLC was reduced from >64 μg/ml to 2 μg/ml in *C*. *tropicalis* after in combination with B-7b. Notably, we observed that FLC and B-7b did not display synergism against FLC-sensitive *C*. *albicans* SC5314 (Table 3).

**Table 3:** MICs of B-7b alone and in combination with FLC against varied yeast strains.

Yeast strains	MIC80(μg/ml)			FICI
	FLC	B-7b	FLC/B-7b	
*C*. *albicans* SC5314	0.5	>64	0.25/8	0.56
*C*. *neoformans* 56992	8	>64	8/1	1.008
*C*. *tropicalis* ATCC20026	>64	>64	2/2	0.031
*C*. *parapsilosis* ATCC 22019	4	>64	2/8	0.563
*C*. *krusei* ATCC2340	64	>64	32/32	0.750
*C*. *glabrata* ATCC1182	8	>64	4/32	0.750

Note: FLC: Fluconazole.

### Functional annotation and pathway analysis

Our previous study suggested that mitochondrial aerobic respiration shift and endogenous ROS augmentation contributed to the synergistic action of FLC + BBR against FLC-resistant *C*. *albicans* [29]. However, our present study did not determined endogenous ROS augmentation involved in the synergistic mechanism of FLC and B-7b (S1 Fig). To get an insight on the underlying synergistic mechanism of FLC and B-7b against FLC-resistant isolates, a comprehensive comparative proteomic analysis was carried out in our study. We identified 2078 proteins in *C*. *albicans* using nano LC-MS/MS (Fig 6A), and performed three grouped comparisons to identify patterns of differential proteins (Fig 6C, 6D and 6E) shown in Fig 6B and S1 Table. The pathway analysis and functional annotation using DAVID for differential proteins overrepresented were summarized in Table 4 and S2 Table, respectively.

**Table 4:** Kyoto Encyclopedia of Genes and Genomes (KEGG) pathways enriched in pairwise comparisons (p<0.5).

Pairwise comparison	KEGG pathways	counts	P value
FLC alone versus Control	Oxidative phosphorylation	23	5.06E-09
	Citrate cycle (TCA cycle)	9	5.46E-04
	Pyruvate metabolism	6	4.32E-03
	Glycolysis / Gluconeogenesis	7	4.75E-03
	Steroid biosynthesis	6	4.10E-02
FLC+8-7b versus Control	Citrate cycle (TCA cycle)	12	4.37E-06
	Oxidative phosphorylation	20	7.36E-06
	Glycolysis / Gluconeogenesis	9	2.78E-04
	Starch and sucrose metabolism	6	3.82E-03
	Steroid biosynthesis	5	9.23E-03
	Alanine, aspartate and glutamate metabolism	6	3.54E-02
	Pyruvate metabolism	5	3.69E-02
FLC+8-7b versus FLC alone	Arginine and proline metabolism	6	3.16E-03
	Oxidative phosphorylation	7	8.39E-03
	Glycolysis / Gluconeogenesis	5	1.91E-02
	Alanine, aspartate and glutamate metabolism	5	1.91E-02
	Protein processing in endoplasmic reticulum	5	3.29E-02

According to the above analysis, the steroid biosynthesis pathway was significantly (p <0.05) enriched in both comparison 1 (FLC treated versus Control sample) and 2 (FLC+B-7b treated versus Control sample) as shown in Table 4 and Fig 7A. Consistent with known protein annotation previously [41,42], proteins including Erg1, Erg3, Erg4, Erg6, Erg251, Erg11, and Erg10 involved in ergosterol biosynthesis were all up-regulated in FLC- and FLC+B-7b-treated sample compared to Control sample (Fig 7B).

**Fig 8:**
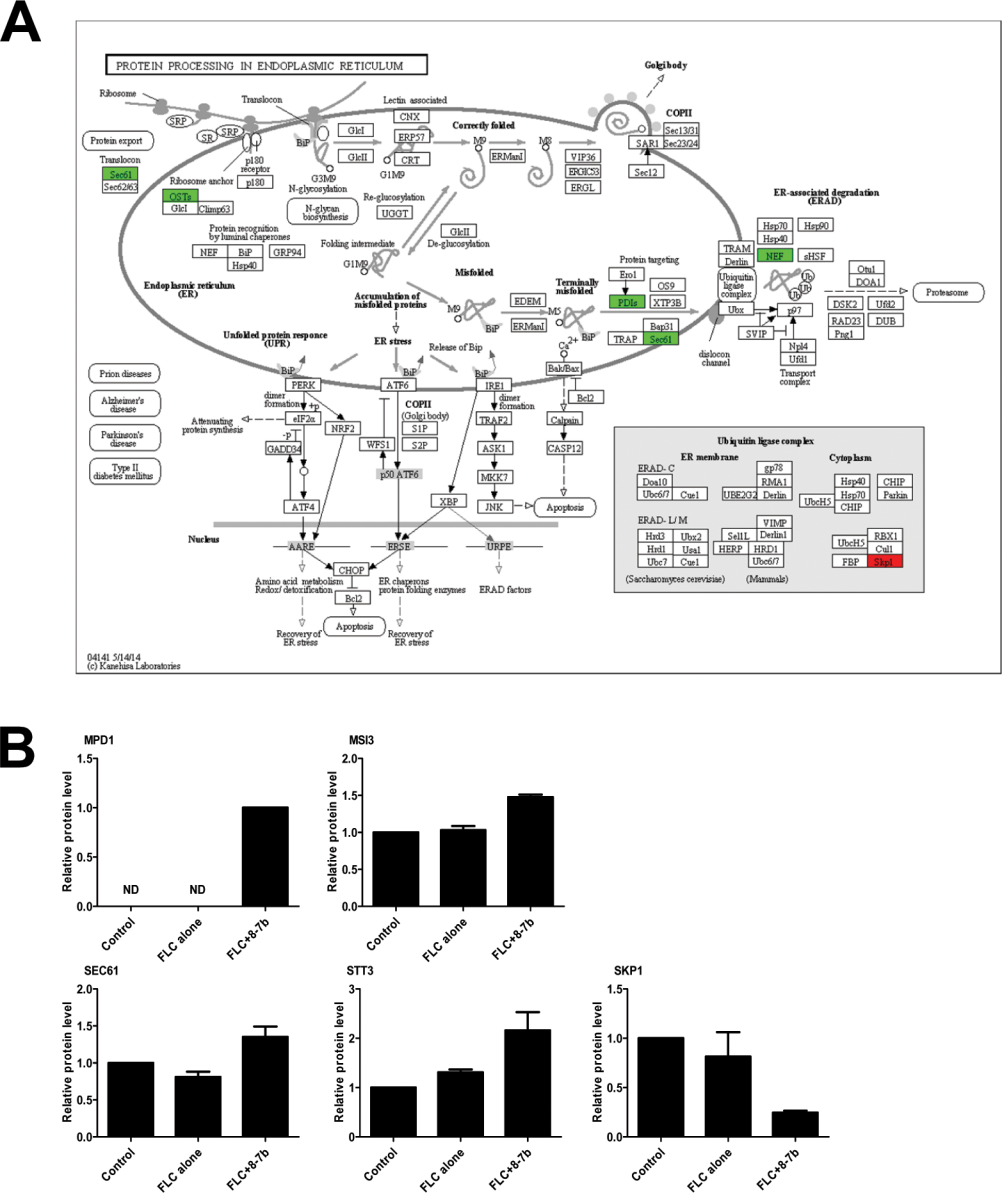
The significant pathway of protein processing in endoplasmic reticulum (p = 0.0329) for differential proteins and the differential protein level in Comparison 3. (A) For each rectangular shape, red shapes indicate down-regulated proteins, while green shapes indicate up-regulated proteins. KEGG pathway analysis figure depicts the role or involvement of a specific protein or a family of related proteins at a particular location in the pathway. (B) The relative protein level of five proteins compared to Control, corresponding to colored rectangular shapes in A, respectively.

**Fig 9:**
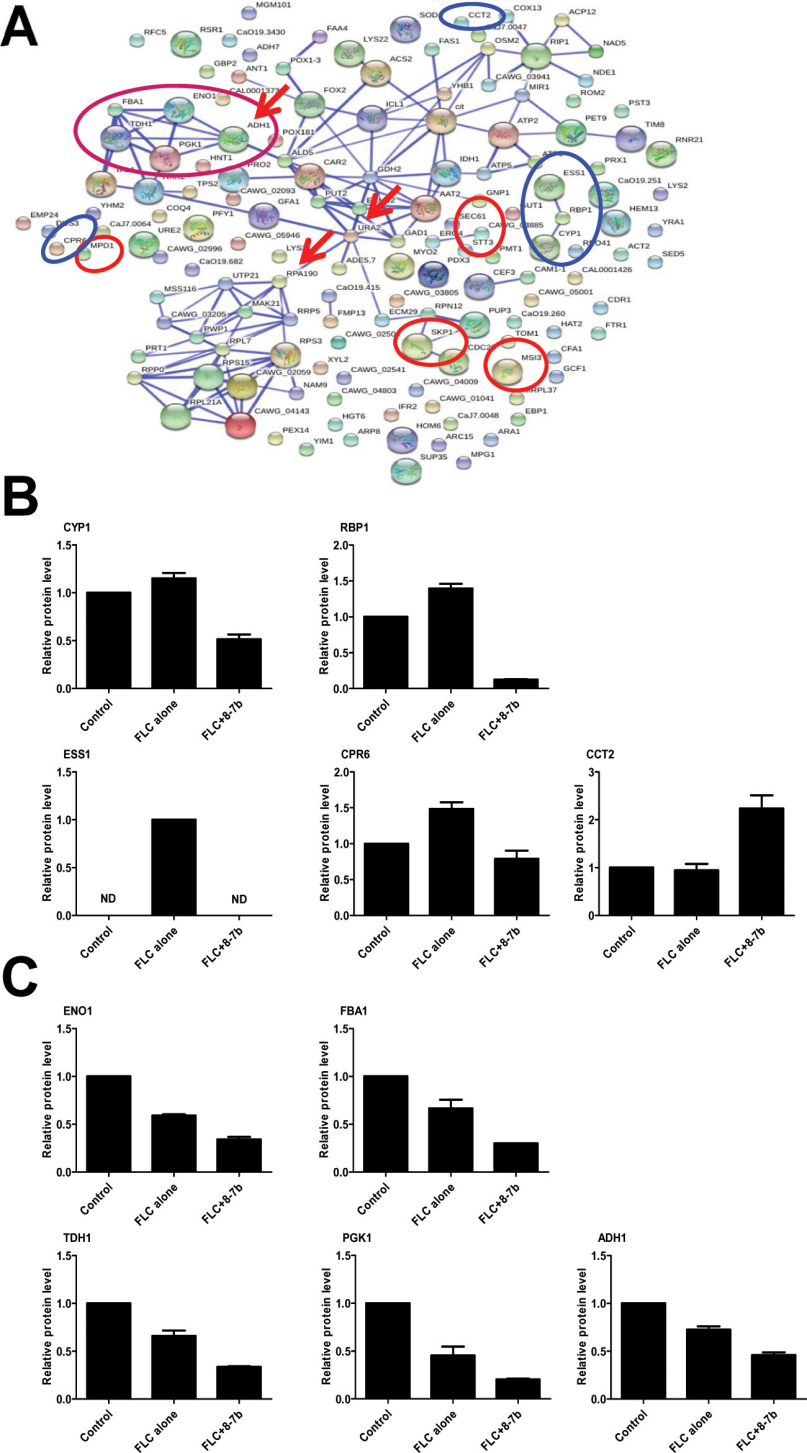
Interaction networks of differential proteins in comparison 3 (confidence score >0.7) and the relative level of differential proteins from different functional categories. (A) The confidence view of all differential proteins altered in *C*. *albicans* cells treated with FLC and B-7b, compared to FLC-treated cells. The strength of the interactions is indicated by the thickness of the connecting lines. Blue, red and purple circles indicate proteins involved in protein folding, protein processing in endoplasmic reticulum and positive regulation of defense response. Red arrows indicate the “hub” proteins. (B) The relative protein level of proteins enriched in protein folding compared to Control. (C) The relative protein level of proteins enriched in positive regulation of defense response compared to Control.

Subsequently, we paid attention to comparison 3 (FLC+B-7b-treated sample versus FLC-treated sample). We observed differential protein sets (Mpd1, Msi3, Sec61, Stt3, Skp1) were enriched in the pathway of Protein processing in endoplasmic reticulum (p = 0.03289, Table 4). The majority of these proteins (4/5) involved in this pathway were up-regulated (Fig 8), while only the protein Skp1, which was putative subunit D of kinetochore protein complex CBF3 and contributed to ubiquitin protein ligase activity in *Saccharomyces cerevisiae* (*S*. *cerevisiae*) [43,44], was significantly down-regulated and had no any interactions with other four proteins as analyzed by STRING (Fig 9A).

Besides, another important finding was that differential proteins from comparison 3 were also significantly classified into some crucial GO categories, such as protein folding and positive regulation of defense response (S2 Table). Compared to FLC-treated sample, as shown in Fig 9B, four out of five proteins (Cyp1, Rbp1, Ess1, Cpr6, Cct2) enriched in protein folding were remarkable down-regulated in FLC+B-7b-treated sample, and three down-regulated proteins (Cyp1, Rbp1, Ess1) showed associations with high confidence score >0.7 (Fig 9A). Particularly, proteins (Eno1, Fba1, Tdh1, Pgk1, Adh1) involved in positive regulation of defense response were all significantly decreased (Fig 9C), and exhibited physical or functional interaction networks with high confidence score >0.8 (Fig 9A).

Interestingly, it was worthy note that Adh1, Ura2 and Rpa190 connected with many partners were dramatically down-regulated (p <0.05, Fig 9A), and became connectors among positive regulation of defense response, amino acid metabolism (arginine, alanine, aspartate, glutamate and proline metabolism, shown in Table 4) and ribosome though in which differential proteins (e.g. Rpl7, Rps15, Rps3 and Rpl21A) were not significantly enriched, suggesting they might be “hub” proteins in *C*. *albicans* cells treated with FLC and B-7b.

## Discussion

Previous researches have demonstrated the mechanism of synergism against FLC-resistant *C*. *albicans* such as: increasing reactive oxygen species (ROS) to promote apoptosis [45], and inhibiting drug efflux pumps to increase intracellular drug concentration [46,47]. For the synergism of FLC and BBR, our previous comparative proteomic analysis suggested that mitochondrial aerobic respiration shift and endogenous ROS augmentation contributed to the synergistic effect of FLC and BBR in FLC-resistant *C*. *albicans* [29].

Based on the above findings, we further investigated the synergistic mechanism of FLC and B-7b against FLC-resistant *C*. *albicans*. Surprisingly, the synergistic action of FLC and B-7b did not lead to the increased generation of endogenous ROS in the study (S1 Fig). We also found that B-7b and FLC did not have synergism against FLC-sensitive *C*. *albicans* (Table 3), and it seems that the synergistic antifungal activity of FLC+B-7b is relevant to FLC resistance. To further understand how FLC and B-7b worked synergistically against FLC-resistant *C*. *albicans*, we employed a comparative proteomic study in FLC-resistant *C*. *albicans* 103 cells untreated or treated with FLC and/ or B-7b. The proteins (such as Erg1, Erg3, Erg4, Erg6, Erg10, Erg24, Erg251 and Erg11) involved in the ergosterol biosynthesis pathway were all up-regulated in comparison 1 or 2 (Fig 7), consisting with previous reports [41,42], while only one of these eight proteins (i.e. Erg4) was significantly changed in comparison 3, suggesting that the combination of FLC and B-7b had very weak or even no effect on the ergosterol biosynthesis pathway. KEGG and GO analysis first indicated that proteins involved in protein processing in endoplasmic reticulum and protein folding were significantly changed, such as remarkable down-regulated protein Cyp1, Rbp1 and Ess1 which are required for protein processing and growth in *C*. *albicans* [48], suggesting the impact of FLC+B-7b on the growth of FLC-resistant *C*. *albicans* could occur through modulation of proteins associated with the protein processing pathway. In addition, according to the GO and PPI networks analysis, we observed that positive regulation of defense response-related proteins (i.e. Eno1, Fba1, Tdh1, Pgk1 and Adh1) were all significantly down regulated (Fig 9C) and co-expressed, and therefore it seemed that the synergistic effect of FLC and B-7b could weaken the cell defense response to drugs.

Adh1, an immunogenic alcohol dehydrogenase, play a role in adherence and response to the extracellular matrix and stimulus [49,50]. In *S*. *cerevisiae*, Ura2 is bifunctional carbamoylphosphate synthetase/aspartate transcarbamylase and catalyzes the first two enzymatic steps in the de novo biosynthesis of pyrimidines [51,52], while it is not verified by direct assays and still uncharacterized in *C*. *albicans*. Rpa190 is yet uncharacterized in *C*. *albicans*, while it is verified to be RNA polymerase I largest subunit A190 in *S*. *cerevisiae* [53]. Therefore, the putative “hub” proteins Adh1, Ura2 and Rpa190 constructing the interconnectivity of three functional categories (i.e. positive regulation of defense response, amino acid metabolism and ribosome), shown in Fig 9A, may play critical roles in the synergistic mechanism of B-7b and FLC against FLC-resistant *C*. *albicans*, and further studies are necessary to be performed.

In conclusion, our study first investigated the synergistic antifungal activity and possible mechanism of novel scaffold of BBR derivatives. The better synergistic activity and much lower cytotoxicity suggested that the representative compound B-7b could serve as a promising lead compound for further research. The results presented here offer the first insights into the synergistic mechanism of FLC and B-7b. Analysis of global protein patterns shift suggested positive regulation of defense response and protein processing involved in the synergism. Further studies to identify the target of B-7b should be carried out.

## Supporting Information

S1 Fig Intracellular ROS generation in *C*. *albicans* 103 cells. Cells were treated or untreated with FLC (64 μg/ml) and/or BBR (16 μg/ml), B-7b (16 μg/ml) for 5 h. *, P<0.05 versus cells treated with FLC (64 μg/ml) alone.
(TIF)

S1 Table The number of different protein identifiers in pairwise comparisons. (XLSX)

S2 Table Significantly enriched gene ontology (GO) terms in each comparison (p <0.05). (XLSX)
