# Supplementary figures and images for: Synergistic Antifungal Activity of Berberine Derivative B-7b and Fluconazole
Source: PLoS One. 2015 May 19;10(5):e0126393. doi: 10.1371/journal.pone.0126393 (PMC4438075; doi:10.1371/journal.pone.0126393)

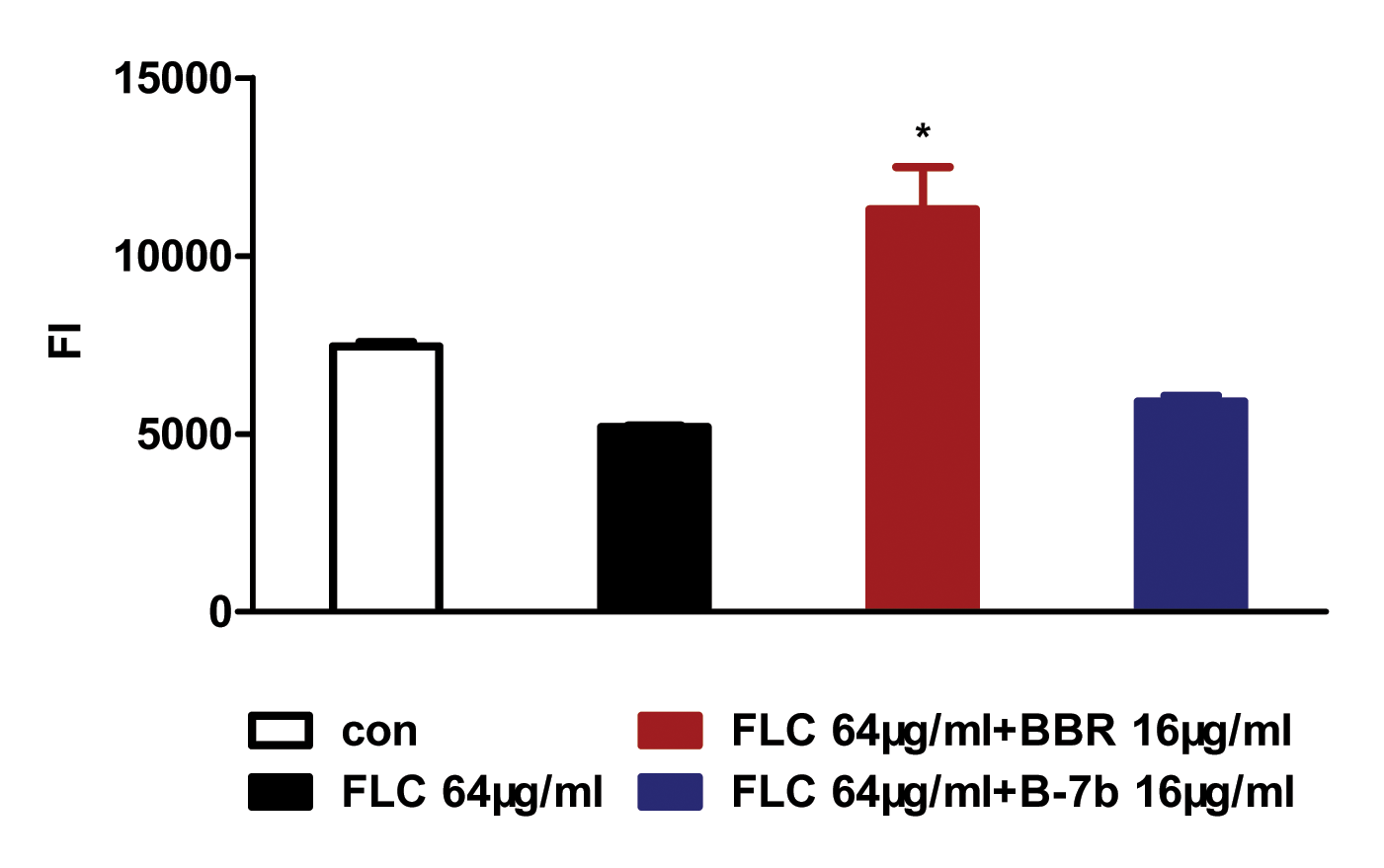

Supplement: S1 Fig — Cells were treated or untreated with FLC (64 μg/ml) and/or BBR (16 μg/ml), B-7b (16 μg/ml) for 5 h. *, P<0.05 versus cells treated with FLC (64 μg/ml) alone. (TIF) [file pone.0126393.s001.tif]
